# Unveiling Antibiotic Resistance, Clonal Diversity, and Biofilm Formation in *E. coli* Isolated from Healthy Swine in Portugal

**DOI:** 10.3390/pathogens13040305

**Published:** 2024-04-09

**Authors:** Adriana Silva, Vanessa Silva, Maria de Lurdes Enes Dapkevicius, Mónica Azevedo, Rui Cordeiro, José Eduardo Pereira, Patrícia Valentão, Virgílio Falco, Gilberto Igrejas, Manuela Caniça, Patrícia Poeta

**Affiliations:** 1 Microbiology and Antibiotic Resistance Team (MicroART), Department of Veterinary Sciences, University of Trás-os-Montes and Alto Douro (UTAD), 5000-801 Vila Real, Portugal; 2 LAQV-REQUIMTE, Department of Chemistry, NOVA School of Science and Technology, Universidade Nova de Lisboa, 2829-516 Caparica, Portugal; 3 Department of Genetics and Biotechnology, University of Trás-os-Montes and Alto Douro (UTAD), 5000-801 Vila Real, Portugal; 4 Functional Genomics and Proteomics Unit, University of Trás-os-Montes and Alto Douro (UTAD), 5000-801 Vila Real, Portugal; 5 IITAA—Institute of Agricultural and Environmental Research and Technology, University of the Azores (UAc), 9700-042 Angra do Heroísmo, Portugal; 6 Centre for the Studies of Animal Science, Institute of Agrarian and Agri-Food Sciences and Technologies, Oporto University, 4049-021 Porto, Portugal; monica.azevedo1297@gmail.com (M.A.);; 7 CIISA—Center for Interdisciplinary Research in Animal Health, Faculty of Veterinary Medicine, University of Lisbon, 1300-477 Lisbon, Portugal; 8 National Reference Laboratory of Antibiotic Resistances and Healthcare Associated Infections (NRL-AMR/HAI), Department of Infectious Diseases, National Institute of Health Dr. Ricardo Jorge, 1649-016 Lisbon, Portugal; 9 Intergados, SA, Av. de Olivença, S/N, 2870-108 Montijo, Portugal; rpgcordeiro@hotmail.com; 10 CECAV—Veterinary and Animal Research Centre, University of Trás-os-Montes and Alto Douro, 5000-801 Vila Real, Portugal; 11 Associate Laboratory for Animal and Veterinary Sciences (AL4AnimalS), 5000-801 Vila Real, Portugal; 12 Laboratory for Green Chemistry (LAQV) of the Network of Chemistry and Technology (REQUIMTE), Universidade do Porto, 2829-516 Caparica, Portugal; valentao@ff.up.pt (P.V.);; 13 REQUIMTE/LAQV, Laboratório de Farmacognosia, Departamento de Química, Faculdade de Farmácia, Universidade do Porto, R. Jorge Viterbo Ferreira, No. 228, 4050-313 Porto, Portugal; 14 Centre for the Research and Technology of Agro-Environmental and Biological Sciences (CITAB), Universidade of Trás-os-Montes and Alto Douro (UTAD), 5000-801 Vila Real, Portugal

**Keywords:** *Escherichia coli*, antimicrobial resistance, food safety, swine farms, multi-resistance, animal production

## Abstract

*Escherichia coli*, a commensal microorganism found in the gastrointestinal tract of human and animal hosts, plays a central role in agriculture and public health. Global demand for animal products has promoted increased pig farming, leading to growing concerns about the prevalence of antibiotic-resistant *E. coli* strains in swine populations. It should be noted that a significant portion of antibiotics deployed in swine management belong to the critically important antibiotics (CIA) class, which should be reserved for human therapeutic applications. This study aimed to characterize the prevalence of antibiotic resistance, genetic diversity, virulence characteristics, and biofilm formation of *E. coli* strains in healthy pigs from various farms across central Portugal. Our study revealed high levels of antibiotic resistance, with resistance to tetracycline, ampicillin, tobramycin, and trimethoprim-sulfamethoxazole. Multidrug resistance is widespread, with some strains resistant to seven different antibiotics. The *amp*C gene, responsible for broad-spectrum resistance to cephalosporins and ampicillin, was widespread, as were genes associated with resistance to sulfonamide and beta-lactam antibiotics. The presence of high-risk clones, such as ST10, ST101, and ST48, are a concern due to their increased virulence and multidrug resistance profiles. Regarding biofilm formation, it was observed that biofilm-forming capacity varied significantly across different compartments within pig farming environments. In conclusion, our study highlights the urgent need for surveillance and implementation of antibiotic management measures in the swine sector. These measures are essential to protect public health, ensure animal welfare, and support the swine industry in the face of the growing global demand for animal products.

## 1. Introduction

Global consumption of animal products is increasing and is expected to increase from 29% to 35% by 2030 and 37% by 2050 in the coming years due to economic growth and urbanization [1,2]. The growing demand for animal products has led to increased animal farming practices, leading to a number of consequences, including defective processing practices and a high risk of contamination by foodborne pathogens [3,4]. Pigs are one of the most consumed livestock in the world, contributing about 33% of the total global meat production [5,6]. As part of management measures to promote weight gain in pigs, antibiotics are used as growth enhancers to prevent the colonization of pathogenic bacteria and the uncontrolled growth of intestinal microorganisms [5,7]. Food-producing animals are considered important reservoirs of foodborne pathogens [3], especially antibiotic-resistant *E. coli* strains. In Europe, a significant fraction of these strains found in food-producing animals exhibit resistance to at least one class of antibiotics. The European Food Safety Authority (EFSA) has reported a concerning trend in *E. coli* strains having decreased susceptibility to fluoroquinolones [8]. The concept of a “high-risk clone” is used to describe bacterial strains that accelerate the transmission of antibiotic resistance. These strains are a source of considerable concern, not only because they pose significant difficulties in treating patients but also because they serve as highly effective vehicles for mobile genetic elements carrying antibiotic-resistant genes, thus accelerating the spread of these genes [9,10]. 

*Escherichia coli* is a commensal bacterium commonly found in the gastrointestinal tract of both humans and animals and it can be introduced after fecal contamination [2,11]. Some strains of *E. coli* are considered harmless species and establish a mutually beneficial relationship with the host, while others are capable of causing disease [8,12]. *E. coli* is associated with a variety of infections, including bacteremia, wound infections, urinary tract infection, and gastrointestinal tract infections [2]. In the pig farming sector, commensal *E. coli* is of great importance and serves as a vehicle for the transmission of multidrug resistance (MDR) traits. MDR traits can spread from contaminated animal products to humans or be introduced directly into the natural environment through fecal matter [13,14]. *E. coli* has become a repository of antibiotic-resistant genes, making it a key indicator for monitoring the prevalence of antibiotic resistance, a role recognized by the World Organization for Animal Health (OIE) [2,15,16]. In addition, *E. coli* also has the ability to transfer genes to other bacterial species, including pathogens [15]. Antibiotic resistance has become a global concern in food-producing animals in recent decades [17]. The use of antibiotics in animal therapy has led to the emergence of antibiotic-resistant bacteria [18], which has important implications for both veterinary and human medicine. This not only limits treatment options but also facilitates the transmission of resistant bacteria from livestock to humans [17,18] through genes transfer in the food chain [19].

*E. coli* is an important infectious agent responsible for a wide range of diseases in pigs and the different pathological manifestations are due to several characteristics, such as virulence factors (adhesins and toxins), resistance genes, and biofilm formation [20]. Penicillin and tetracycline are the most used antimicrobial agents in livestock; however, high resistance to ampicillin, sulfamethoxazole, and trimethoprim was prevalent among indicator *E. coli* collected at slaughterhouses [20]. *E. coli* has numerous genes that encode for resistance to β-lactams, with some conferring resistance only to ESBLs, which can inactivate penicillin and aminopenicillins. An increase in the prevalence of ESBLs compromises treatment effectiveness and increases morbidity and mortality. ESBL confers resistance to β-lactam antibiotics such as penicillin, first-, second-, and third-generation cephalosporins, and aztreonam. However, cephamycins and carbapenems are not effective [4,20].

Although *E. coli* is less common in healthcare-associated biofilms, it remains a potential cause of sepsis. Biofilm formation is considered a pathogenic factor that plays an important role in antimicrobial resistance, leading to reduced permeability and contributing to reduced susceptibility to antimicrobial agents and invasive infections [21,22]. The ability to form biofilms also facilitates the spread of resistance genes in the environment, and bacteria can exchange genetic elements to facilitate the dissemination of resistance traits [23]. The increasing prevalence of MDR *E. coli* strains capable of biofilm formation necessitates the development of new, effective, and safe antimicrobial agents to combat resistant bacteria, especially those producing ESBLs and with carbapenem resistance [24]. Several clones of *E. coli* have been identified, some of which can be classified as pandemic strains and MDR, as is the case of ST131, ST10, ST69, ST95, and ST73. It is important to establish the clonal relationship between strains from different hosts and diseases to assess the risk of zoonotic infections [25]. 

Swine are considered one of the most important farm animals in terms of number and biomass and in Portugal as characterized by its large-scale pig farms. Numerous studies have addressed the antimicrobial resistance of *E. coli*, but there have been fewer studies in Portugal on the correlation between antimicrobial resistance, virulence factors, genetic diversity, and whole genome sequence and biofilm formation of *E. coli* in healthy pigs. Our objective was to investigate the prevalence of *E. coli* in healthy pigs from various farms across central Portugal, where there is a high concentration of pig farms. This work provides information on the antimicrobial resistance, virulence, genetic diversity, and biofilm formation of *E. coli* populations in healthy pigs from various farms across central Portugal and highlights the potential implications in public health and in agricultural sector.

## 2. Materials and Methods

### 2.1. Samples Collection and Bacterial Isolates

**Figure 1:**
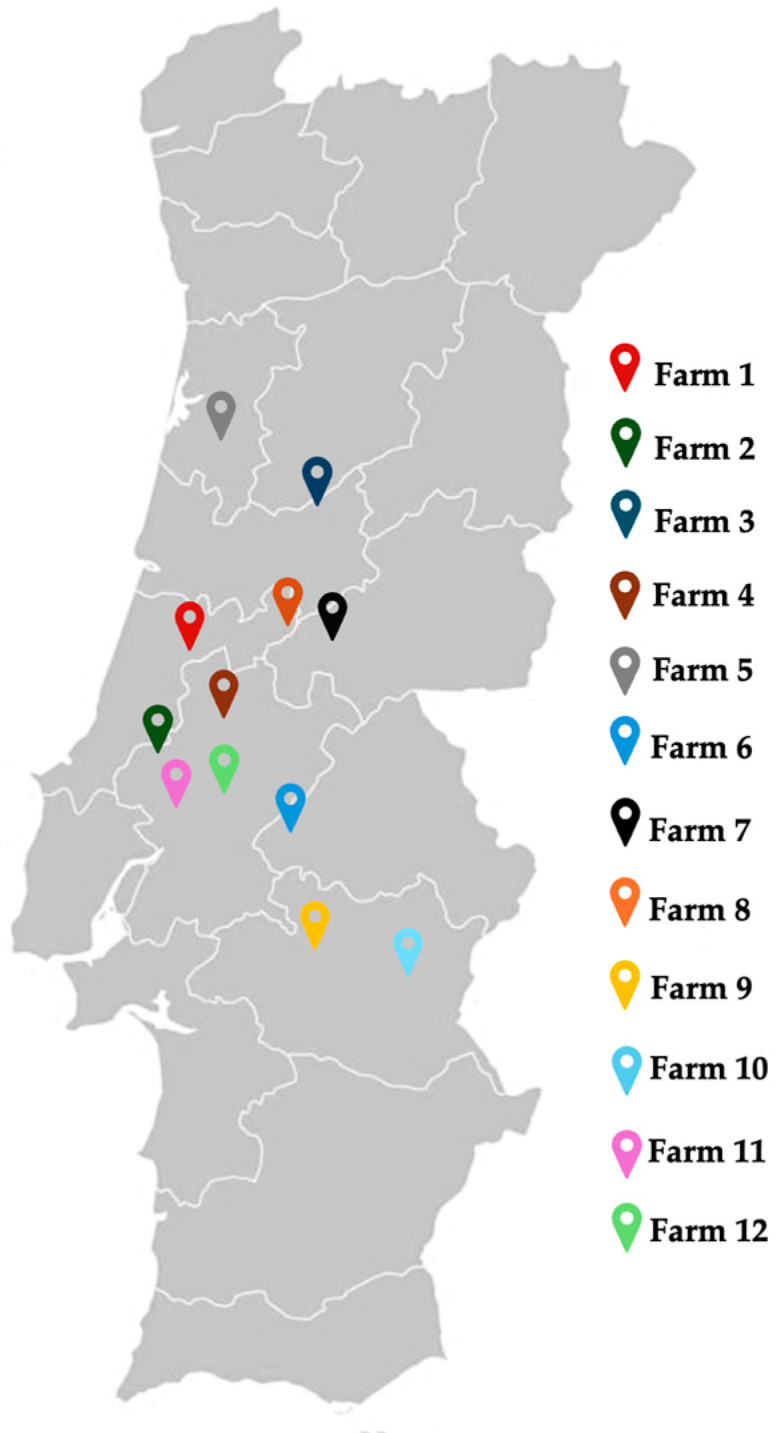
Locations of sampled pig farms across the center of Portugal.

A total of 65 fecal samples were collected from twelve different healthy pig farms located across the center of Portugal: 10 breeding pig farms and 3 fattening pig farms. Nine farms were exclusively breeding pigs and two were fattening pigs; however, one pig farm was both a breeding and fattening farm (Figure 1). From the twelve different healthy pig farms, farms 1, 5, 8, 7, 2, 4, 6, 7, and 9 each yielded 8 samples. Farms 10 and 11 contributed 3 samples each, and farm 12 provided two fecal samples, ensuring a statistically representative sample count. Fecal samples were collected, stored at 4 °C, and transferred to the MicroART-Microbiology and antibiotic resistance team laboratory in University of Trás-os-Montes and Alto-Douro (UTAD) located in Vila Real (Portugal) for processing, not exceeding a maximum of 24 h.

### 2.2. Isolation and Identification of Escherichia coli

From each fecal sample, a 5 g aliquot was homogenized and diluted in Brain Heart Infusion (BHI) broth (LiofilChem, Via Scozia, Italy) under aerobic conditions, and incubated for 24 h at 37 °C. After this process, samples were placed on Eosin–Methylene Blue (EMB) agar plates (Oxoid) and on MacConkey agar plates (Conda, Spain) and incubated for 24 h at 37 °C. Presumptive colonies exhibiting morphological characteristics of *E. coli* were recovered (one colony per sample) and identified using a standard biochemical test—the IMViC reactions (Indol, Methyl-red, Voges-Proskauer, and Citrate). Matrix-Assisted Laser Desorption/Ionization Time-of-Flight Mass Spectrometry (MALDI-TOF MS, MALDI Biotyper^®^, Bruker Daltonik, Bremen, Germany) was applied in this study to confirm the identification of the bacterial isolates at the species level. *E. coli* isolates were kept at −80 °C until further characterization.

### 2.3. Antibiotic Susceptibility Testing

Antibiotic susceptibility was assessed using the Kirby–Bauer disk diffusion method and consists of using colony suspension and adjusting the turbidity of this suspension to a 0.5 McFarland standard, corresponding approximately to 1–2 × 10^8^ CFU/mL on Muller–Hinton agar, according to the European Committee on Antimicrobial Susceptibility Testing (EUCAST, 2021) guidelines [26]. A panel of 16 antibiotics (µg/disk), relevant to both human and animal health, was used and the diameter of the zones where complete inhibition occurred was measured: ampicillin (10 μg), amoxicillin plus clavulanic acid (AMC) (20 + 10 μg), cefoxitin (30 μg), cefotaxime (30 μg), ceftazidime (30 μg), aztreonam (30 μg), imipenem (10 μg), gentamicin (10 μg), amikacin (30 μg), tobramycin (10 μg), streptomycin (10 μg), nalidixic acid (30 μg), ciprofloxacin (5 μg), sulfamethoxazole-trimethoprim (SXT) (25 μg), tetracycline (30 μg), and chloramphenicol (30 μg). The plates were incubated aerobically for 24 h at 35 °C. Based on the diameter of the inhibition zone around the antibiotic disks, isolates were classified as susceptible or resistant towards each of the antibiotics under study, in accordance with the EUCAST tables. Screening for phenotypic ESBLs (extended-spectrum β-lactamase) was performed by the double-disc diffusion synergy test (using cefotaxime, ceftazidime, and AMC disks) [27]. One isolate per fecal sample was selected for further studies and *E. coli* ATTC 25922 served as the positive control. 

### 2.4. Characterization of Antimicrobial Resistance Gene and Virulence Genotyping of E. coli

Genomic DNA was obtained from *E. coli* isolates extraction by the boiling method. Briefly, a single colony grown overnight was suspended in 1 mL of MilliQ water, then boiled for 8 min to disrupt the cell walls, centrifuged at 12,000 rpm for 2 min, and the pellet was discarded. DNA concentration was evaluated using a Nanodrop spectrophotometer and used as the DNA template for all PCR reactions. Each 50 μL PCR mixture contained 5 μL PCR buffer, 1 μL 2 mM dNTP, 1 μL of each primer, 30.2 μL sterile distilled water, 0.3 μL Taq DNA polymerase, 1.5 μL of MgCl_2_, and 10 μL DNA template. The presence of *bla*CTX-U, *bla*TEM, *bla*IMP, *bla*SHV, *bla*OXA, *bla*VIM, and *amp*C was tested by PCR assay in 39 isolates. The presence of genes encoding resistance to non-beta-lactams was studied by PCR. The presence of genes associated with resistance to tetracycline (*tetA*, *tetM*, and t*etB*), sulphonamides *(sul2* and *sul3*), streptomycin (*str*A and *str*B), chloramphenicol (*cmlA*), aminoglycosides (*ant(2*), *aph(3*), *aac(3)-*II, *aac(3)-*IV, and *aad*A1), and quinolones (*qnr*S, *par*C, and *aac(6′)-Ib-cr*) was determined for all resistant *E. coli* isolates. The presence of *int1* and *int2* genes encoding class 1 and class 2 integrases, respectively, and its variable region (*RVint1* and *RVint2*) was also examined by PCR [28]. *E. coli* isolates were screened by PCR assay to detect the presence of genes encoding the following virulence factors: *fimA* (type 1 fimbriae), *papGIII* (adhesin *PapG* class III), *hlyA* (hemolysin), *cnf1* (cytotoxic necrotizing factor), *papC* (P fimbriae), *aer* (aerobactin iron uptake system), *eae* (Intimin), and *bfp* (Type IV bundle forming pili) [28]. 

### 2.5. Whole Genome Sequencing Analysis

Seven strains (AS3, AS17, AS31, AS33, AS34, AS42, and AS46) were selected to be further studied by whole genome sequencing (WGS) and were classified based on their resistance profile, and one representative of each group was selected. Strains AS3 and AS34 exhibit resistance to seven different classes of antibiotics. Strains AS17 and AS46 are resistant to five different classes of antibiotics, with AS17 resistant to quinolones and AS46 resistant to chloramphenicol. AS31 and AS33 are resistant to six different classes, with the difference that AS31 is resistant to chloramphenicol, while AS33 is resistant to quinolones. AS42 demonstrates resistance to four classes of antibiotics. A comprehensive whole genome analysis was conducted using the TORMES v1.3.0 bioinformatics pipeline. To assess the genomes for acquired antibiotic resistance genes and VFs, ResFinder and VirulenceFinder v2.0 (Center for Genomic Epidemiology, Technical University of Denmark, Lyngby, Denmark) servers were used. The Comprehensive Antibiotic Resistance Database (CARD) was used to search the genome for acquired antibiotic resistance genes. MLST v 2.0.4, PlasmidFinder v2.1, and SerotypeFinder 1.1 (Center for Genomic Epidemiology) were used with default settings to determine MLST type, plasmid types, and serotypes of the isolates. Additionally, identification of the major phylogenetic groups (A, B1, B2, or D) of *E. coli* isolates was determined by PCR using a combination of three genes (*chu*A, *yja*A, and *Tsp*E4.C2), as previously described by Clermont et al. [29].

### 2.6. Biofilm Formation

Biofilm formation assay was performed according to a previously described protocol, with some modifications. Briefly, two colonies were transferred from the fresh culture into tubes containing 3 mL of Tryptic Soy Broth (TSB, Oxoid, Basingstoke, UK) and then incubated at 37 °C for 16 ± 1 h with continuous shaking, at 120 rpm, using a shaker incubator (ES-80 Shaker, Grant Instruments, Cambridge, UK). After this incubation period, the bacterial suspension was standardized to an optical density equivalent to 1 × 10^6^ colony-forming units. Then, 200 μL of the bacterial suspensions from each different isolate were added to individual wells of a 96-well, flat-bottom microplate. As a positive control, *E. coli* ATTC 25922 was included in all plates, while fresh, sterile medium, without bacterial inoculation, was used as a negative control. The plates were then incubated at 37 °C for 24 h without shaking. For each experiment, seven technical replicates were prepared, and each was performed in triplicate. Biofilm mass was assessed by the Crystal Violet (CV) staining method, following the procedure described by Peeters et al. (2008) [30], with some modifications. After the incubation period, each well was washed twice with 200 μL of distilled water to remove non-adherent bacterial cells. The plates were then left to air dry at room temperature for approximately 2 h. To fix the microbial biofilm, 100 μL of methanol (VWR International Carnaxide) were added to each well, and allowed to react for 15 min. Then, methanol was removed, and the plates were once again air-dried at room temperature for 10 min. Then, 100 μL of 1% (*v*/*v*) CV solution was added to each well and allowed to sit for 10 min. Then, excess CV solution was removed by washing the plates twice with distilled water. To dissolve CV, 100 μL of 33% (*v*/*v*) acetic acid was added, and the absorbance was measured at 570 nm using a microplate reader (Bio Tek elX808U, Winooski, VT, USA) [30]. For each isolate under study, biofilm formation results were presented as a percentage of the results obtained for the reference strain.

## 3. Results

### 3.1. E.coli Isolation

From January 2021 to October 2021, 59 pig fecal samples were received and sampled for isolation from 12 different pig farms, from which 46 were positive for *E. coli*, subsequently being identified by biochemical and MALDI-TOF tests. Of these, a total of 44 *E. coli* isolates were obtained from samples collected from breeding pigs and two *E. coli* isolates were gathered from fattening pigs. Of these 44 *E. coli* isolates collected from breeding pigs, 12 *E. coli* isolates originated from gestation pens (29.5%, 13/44), 12 were obtained from gestation parks (31.8%, 14/44), 11 were sourced from maternity pens (25%, 11/44), 1 was isolated from nulliparous pigs (2.27%, 1/44), and 5 were derived from rearing pens (11.36%, 5/44). 

### 3.2. Antibiotic Resistance Profile of E. coli Isolates

**Figure 2:**
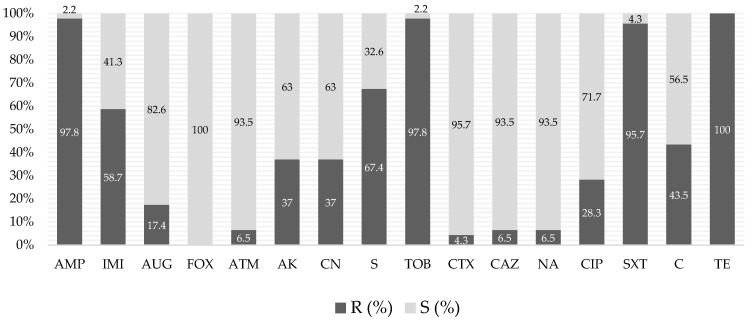
Antibiotic susceptibility pattern of *Escherichia coli* isolates from different swine droves, including breeding and fattening pigs (January 2021–October 2021). R—resistant; S—susceptible. IMI—imipenem; AUG—amoxicillin-clavulanic acid; FOX—cefoxitin; ATM—aztreonam; AMP—ampicillin; AK—amikacin; CN—gentamicin; S—streptomycin; TOB—tobramycin; CTX—cefotaxime; CAZ—ceftazidime; NA—nalidixic acid; CIP—ciprofloxacin; SXT—trimethoprim-sulfamethoxazole; C—chloramphenicol; TE—tetracycline.

Antibiotic resistance in *E. coli* isolates from the swine droves (n = 46) was detected in all 16 antibiotics tested (Figure 2). We found a high frequency of resistance to tetracycline (100%), ampicillin (97.8%), tobramycin (97.8%), and trimethoprim-sulfamethoxazole (95.7%). None of the isolates were resistant to cefoxitin and they had a high rate of susceptibility to amoxicillin-clavulanic acid (82.6%), aztreonam (93.5%), cefotaxime (95.7%), ceftazidime (93.5%), and nalidixic acid (93.5%). Notable differences in the resistance patterns were observed when looking at each compartment separately. Seven different classes of antibiotics were tested, including beta-lactams, aminoglycosides, cephalosporins, quinolones, sulfonamides, miscellaneous agents, and tetracyclines. Multidrug resistance (MDR), meaning resistance to at least three or more antibiotic classes, was demonstrated in all *E. coli* isolates. Specifically, 9 isolates were resistant to four distinct antibiotic classes, 18 isolates were resistant to five diverse antibiotic classes, 13 isolates were resistant to six different antibiotic classes, and 6 isolates were resistant to seven distinct antibiotic classes.

### 3.3. Molecular Characterization and Whole Sequence Genome

Table 1 provides an overview of the *E. coli* isolates, including their origin, farm compartment studied, antimicrobial resistance profiles (phenotype and genotype), phylogroups, integrons, and virulence factors associated with each isolate. Among the 39 *E. coli* isolates analyzed, several antibiotic resistance genes were identified, and the most common resistance genes in this study are listed next. First, *ampC*, which encodes *amp*C beta-lactamase, and confers resistance to extended-spectrum cephalosporins and ampicillin, was present in a considerable proportion (84.61%) of the isolates. Second, the *sul*3 gene, associated with resistance to sulfonamide antibiotics, was found in 58.97% of the isolates. In addition, *bla*TEM, responsible for beta-lactamase production and resistance to penicillin and to some cephalosporins, was present in approximately 53.84% of the *E. coli* isolates. Several other resistance genes were also detected in our study, with lower prevalences. These genes include *tet*A (25.64%) and *tet*B (41.02%), suggesting resistance to tetracycline antibiotics, *aac(6)-*Ib (30.76%), *acc(3)-*II (25.64%), and *acc(3)-IV* (38.46%), all of which are associated with aminoglycoside resistance. *bla*IMP, associated with beta-lactam resistance, was also identified (23.07%), as well as *par*C (23.07%) and *qnr*S (2.56%), both associated with quinolone resistance. Furthermore, integrons were found in many different isolates, suggesting the possibility of their involvement in rearrangement of gene cassettes, and on the development of antibiotic resistance. Genes associated with virulence factors were widespread and were found in all *E. coli* isolates in this study. Identified virulence genes included *fim*A (100%), *bfp* (41.02%), *aer* (5.12%), *cnf1* (15.38%), and *pap*C (2.56%).

Among the seven isolates studied by WGS, the MLST type showed a variety of antimicrobial resistance profiles, phylogroups, virulence factors, MLST type, O-serotype, and plasmid replicons (Table 2). All the *E. coli* isolates harbored at least one **β**-lactamase gene, with *blaTEM-1B* (71.42%) as the most prevalent variant followed by *bla*TEM-1A (28.57%). Other *blaTEM* variants were found and other detected type were *bla*LAP-2. Most of the *E. coli* strains harbored a genetic region containing an AmpC-promoter (57.14%). A wide variety of antimicrobial resistance genes were identified and since each gene affects the action of antibiotics in a different way, they can affect different targets, such as antibiotic efflux (*H-NS*; *evgA*; *evgS*; *TolC*; *AcrE*; *AcrS*; *msbA*; *emrB*, *emrA*; *acrB*; *acrD*; *Escherichia coli acrA*; *marA*; *mdtG*; *mdtE*; *YojI*; *cpxA*; *gadX*; *mdtO*; *msbA*; *mdtP*); antibiotic target alteration (*PmrF*; *eptA*; *bacA*), antibiotic inactivation (*ampC beta-lactamase*; *aadA2*; *aadA*; *TEM-1*; *LAP-2*), reduced permeability to antibiotics (*marA*), antibiotic target protection (*QnrS1*), and antibiotic target replacement (*sul2*; *dfrA12*; *sul3*). Resistance mechanisms to non-β-lactam antibiotics were identified amongst the selected *E. coli* isolates, including determinants against aminoglycosides (*aph(6)-Id*; *aph (3″)-Ib aadA2* and *aadA1*), chloramphenicol (*cmlA1*), fluoroquinolones (*qnrB19* and *qnrS1*), sulfonamides (*sul2* and *sul3*), tetracyclines (*tet(A*) and *tet(M*)), and trimethoprims (*dfrA1*). All carried the *tet(A*), *sul*, and *dfrA* genes which confer resistance to tetracycline, sulfonamides, and trimethoprim-sulfamethoxazole. Several *E. coli* isolates contained chromosomal mutations in *gyrA*, *gyrB*, *parC*, and *are* that confer fluoroquinolone resistance. Mutations in *folP* confer resistance to sulfonamides. Other mutations were also found in *pmrA*; *pmrB*; *23S*, *rrsC*; *16S-rrsH*, and *гpoB.*

The seven *E. coli* isolates showed a wide array of virulence genes, including iron acquisition systems (*fyuA*, *iroN*, *irp2*, *iucC*, *iutA*, and *sitA*), protectins (*kpsM*), serum resistance (*iss* and *traT*), adhesins (*csgA*, *fdeC*, *fim*, and *pap*), and toxins (*astA*). Six different sequence types (STs) were identified: ST101, ST5229, ST48, ST5757, ST10, and ST1147. These belong to two different phylogenetic groups, B1 and A. Regarding O-serotypes, all strains exhibited different serotypes, as shown in Table 2. The *IncFI* plasmid type was observed to be the most prevalent. Additionally, the sequences were analyzed in PathogenFinder and all were classified as potential human pathogens.

### 3.4. Biofilm Formation

**Figure 3:**
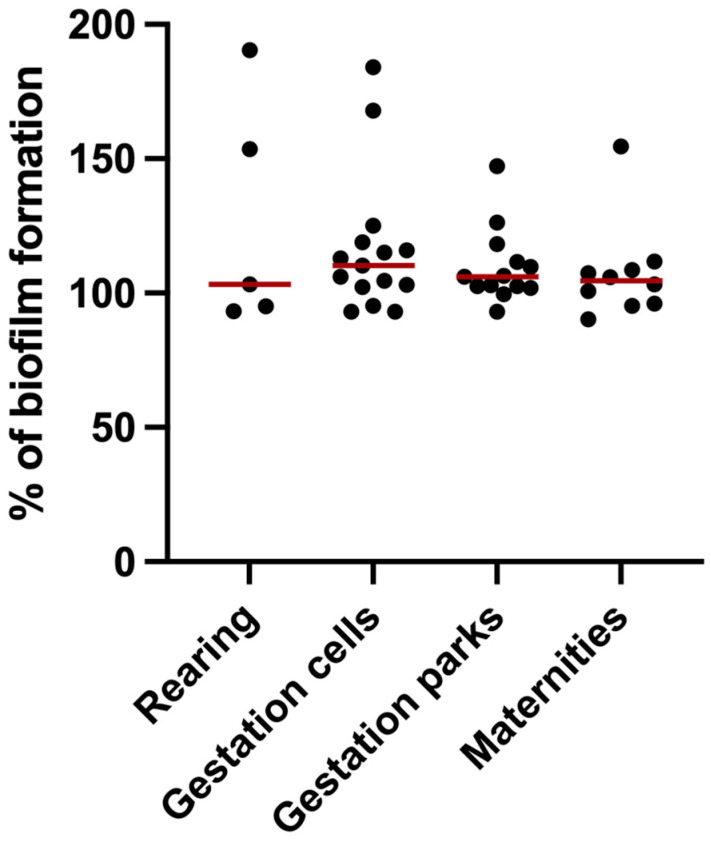
Biofilm formation capacity of *E. coli* strains isolated from different swine herds and compartments. The symbols represent the biomass mean of the biofilm formed in independent tests of the individual isolates. The red lines represent the average of biofilm mass formed by all isolates.

Biofilms were produced by all strains isolated from the different swine droves under study. Results were normalized to *E. coli* ATCC 25922. Figure 3 illustrates biofilm formation by each isolate, organized according to the farm compartment from which they came (rearing, gestation cells, gestation parks, and maternities). *E. coli* isolated from gestation cells had the highest biofilm production ability, above that found in gestation parks, rearing, maternities, nulliparous, and pig fattening compartments. The isolates that produced less biofilm mass were those obtained from pig rearing compartments. Isolates from nulliparous and fattening pigs were excluded due to insufficient sample sizes or non-representative isolates (n = 1 and n = 2, respectively).

## 4. Discussion

Antibiotic usage in swine production is a complex problem, with animal health, animal welfare, and economic implications [31]. Most antibiotics used in swine husbandry are classified as critically important antimicrobials (CIAs) for human therapeutic applications. Furthermore, there is an overlap between antibiotics categorized as CIAs and the list of critically important veterinary antibiotics (VCIA) by the World Organisation for Animal Health (OIE) [32]. Seven of the antibiotics included in our work were part of both the CIA and the VCIA lists (ampicillin, amoxicillin-clavulanic acid, amikacin, gentamycin, streptomycin, tobramycin, and ciprofloxacin), four were included only in the CIA list (ceftazidime, cefotaxime, aztreonam, and imipenem), a few were only in the highly important antibiotics (HIA) list of the WHO (cefoxitin, chloramphenicol, and sulfamethoxazole-trimethoprim), whereas tetracycline is part of both the HIA and VCIA lists. Our results showed concerning rates of resistance to several critical and highly important antibiotics in human and veterinary medicine—tetracycline (100%), ampicillin (97.8%), tobramycin (97.8%), sulfamethoxazole-trimethoprim (95.7%), imipenem (58.7%), streptomycin (67.4%), gentamycin (37.0%), amikacin (37.0%), and chloramphenicol (43.5%). Nevertheless, some broad-spectrum beta-lactam antibiotics (cefoxitin, cefotaxime, and ceftazidime), as well as nalidixic acid, remain effective, with low rates of phenotypic resistance. Several other studies on swine in different parts of the world also describe widespread resistance to ampicillin, aminoglycosides [13,32], tetracyclines [17], sulfamethoxazole-trimethoprim [9,10], and chloramphenicol [17], supporting the concept that similar phenotypic resistance patterns may be present on a global scale. However, in a survey on three different farms, refs. [5,6] revealed a distinct pattern of antibiotic resistance. In their study, *E. coli* isolates exhibited a high rate of resistance to ampicillin (73.5%), chloramphenicol (52.9%), and ciprofloxacin (52.9%), but there was a group of antibiotics, which included aztreonam, cefotaxime, and imipenem, that showed considerably higher efficacy against the studied strains than in our study. Multidrug resistance (MDR) is a significant concern in the context of antibiotic resistance in *E. coli* strains found in pigs and other livestock [3,4]. In our study, all *E. coli* isolates showed MDR, with some being resistant to up to seven different antibiotic classes. Several other studies, from different regions of the world, have consistently reported high MDR rates among *E. coli* isolates associated with pigs, which often exhibit resistance to a broad spectrum of antibiotics, including commonly used ones, such as penicillin and tetracyclines [33]. In contrast, a rate of only 7% MDR was found among swine isolates in a study conducted in the UK [34].

Our study revealed a complex landscape of antimicrobial resistance genes among the *E. coli* isolates, with the most prominent gene, *amp*C, present in 86.95% of the isolates, but determinants of sulfonamide (*sul2* and *sul3*), tetracycline (*tet*A and *tet*B), aminoglycoside (*aac(6)-*Ib, *aph(3*), *acc(3)-*II, *acc(3)-*IV)*,* chloramphenicol (*cml*A), beta-lactam (*bla*TEM, *bla*IMP), and quinolone (*par*C and *qnr*S) resistance were also found. The seven *E. coli* isolates analyzed through WGS also exhibited diverse antimicrobial resistance genes, all isolates had at least one β-lactamase gene, with *bla*TEM-1B being the most prevalent variant. Most had an *ampC*-promoter genetic region and resistance to non-β-lactam antibiotics was also observed, with determinants against aminoglycosides, chloramphenicol, fluoroquinolones, sulfonamides, tetracyclines, and trimethoprim. A study in Peru, refs. [5,6] also identified aminoglycoside, fluoroquinolone (*qnr*B1 and *qnr*S1 genes), tetracycline (*tet*A, *tet*B, and *tet*M), chloramphenicol (*cml*A1), and sulfonamide resistance determinants (*sul2* and *sul3*). The prevalence of *sul2*, *sul3*, and *cml*A1 in isolates from pig farming suggests that resistance to these latter antibiotics is common in swine populations . A study on Thai swine farms [35] revealed that isolates from piglet feces had the highest numbers of antimicrobial resistance genes, and identified eight antimicrobial resistance determinants (*bla*TEM, *tet*A, *drf*A12, *sul3*, *cml*A, *aaad*A1, *Sul2*, and *cat*B), linked to trimethoprim, sulfonamide, aminoglycoside, and tetracycline resistance among these isolates [35]. A study in pigs aged 19–30 days [36] reported that sulfonamide-resistance genes were frequent, with *sul*3 present in 60%, and *sul*2 in 45% of the strains [36]. Both our study and Reid’s highlighted the frequency of sulfonamide-resistance genes, particularly beta-lactamase genes, especially *bla*TEM, contributing to antibiotic resistance against penicillin and specific cephalosporins. However, Reid et al. (2017) [36] reported a lower prevalence (47%) than in our study (60.87%). Nevertheless, both studies found a consistent trend in antibiotic resistance genes among *E. coli* strains.

The MLST analysis by WGS of seven selected *E. coli* strains revealed diverse resistance profiles, strains AS3 and AS34 showed resistance to seven antibiotic classes, while AS17 and AS46 were resistant to five classes, with AS17 being resistant to quinolones and AS46 to chloramphenicol. AS31 and AS33 were resistant to six classes, and AS42 showed resistance to four classes. Six distinct STs were identified: ST101, ST5229, ST48, ST5757, ST10, and ST1147. These STs showed resistance to different antimicrobial classes with the presence of detected antimicrobial-resistant genes, more specifically, **β**-lactamase gene (*bla*TEM) and non-**β**-lactamase genes. Phylogenetic group A was the most prevalent (60.8%), followed by group B1 (30.4%) and group D (8.69%). Studies revealed the genetic diversity and high-risk *E. coli* lineages in swine populations worldwide [3,4] highlighting the need for understanding *E. coli* genetic diversity. 

ST10, a pandemic strain among human ESBL/AmpC-producing ExPEC clones [37,38], has been found in pig samples in the UK [34], in Switzerland [39], and in Australia [40]. Studies show that this clone showed resistance to multiple antimicrobial classes. Our study found two isolates (AS31 and AS46) from clone ST10, a member of the CC10 clonal complex. These isolates belonged to different phylogenetic groups and showed resistance to six and five classes of antibiotics, respectively. Both strains carried the *bla*TEM gene, although the variants were different, AS31 contained *bla*TEM-1A and *bla*LAP, while AS46 contained *bla*TEM-1B. Moreover, they shared common resistance genes such as *aadA*, *aph*(3), *sul3*, *tetA*, and *dfrA*. These genes confer resistance to antibiotics important in both human and veterinary medicine, including aminoglycosides, sulfonamides, tetracyclines, and trimethoprim. When the resistance mechanisms of these two strains are analyzed, we find that both are very similar; however, AS31 has a quinolone resistance protein (*qnr*) that affects antibiotic target protection. Regarding the other resistance genes that were detected by these two strains, the mechanisms that affect them are the same, such as genes that affect antibiotic efflux, antibiotic target alteration, antibiotic inactivation, reduced permeability to antibiotics and antibiotic target replacement. Both strains were also classified as human pathogen strains by PathogenFinder. ST101, a prominent member of CC101, is a significant virulence and multidrug resistance isolate in human ESBL/AmpC-producing ExPEC infection due to its resistance to multiple antibiotics and its phylogenetic group B1 virulence-associated genes [37]. In our study, one isolate, AS42, belongs to this sequence type. It exhibits resistance to four different classes of antibiotics and possesses a variety of resistance genes with multiple resistance mechanisms. These mechanisms include genes affecting antibiotic efflux, reduced permeability to antibiotics, antibiotic inactivation, and antibiotic target replacement. ST48 is associated with a potential zoonotic risk [41]. In our study, ST48 strains were classified as group A and exhibited resistance to antibiotics from five classes. The resistance genes of this strain contribute to antibiotic resistance through mechanisms such as antibiotic efflux, antibiotic target alteration, reduced permeability to antibiotics, antibiotic inactivation, and antibiotic target replacement. A study in Australia [40], identified ST48 in both piglets and sows, being more common in piglets. Both strains had identical antimicrobial resistance genes profiles [40]. The presence of antibiotic resistance genes in ST10, ST101, and ST48, underscores the need for comprehensive surveillance, stronger antibiotic supervision, rational antibiotic use, promotion of alternative antibiotics and effective antimicrobial stewardship in swine production. The widespread use of antibiotics in livestock is accelerating the spread of resistant bacteria and raising serious concerns for human health and animal medicine, as infections with MDR strains can limit treatment options [2]. 

Biofilm formation in *E coli* is a survival strategy, granting resistance to antibiotics and disinfectants, but poses challenges for eradication and control, especially with the increasing prevalence of MDR strains [24,42]. Furthermore, biofilm-producing *E. coli* may have the ability to create complex structures on mucosal surfaces, facilitating gut colonization [23]. The present study investigated biofilm formation in *E. coli* strains isolated from different swine compartments. Compared to the reference strain, *E. coli* ATCC 25922, the results showed considerable heterogeneity in biofilm production according to the source of the isolates. The strains from gestation cells exhibited the highest biofilm-forming capacity, above those from gestation parks, rearing environments, maternities, nulliparous, and fattening pigs. The strains isolated from rearing pigs displayed the lowest biofilm mass. These results may indicate the need to reinforce the sanitary void and sanitation practices in gestation cells, to avoid biofilm buildup. Numerous investigations have been conducted to assess biofilm formation in swine isolates throughout the world [24], with results that point to a high incidence of strong biofilm producers, ranging from a third to most of the isolates. Overall, these studies demonstrate *E. coli* strains from various regions and environments exhibit high biofilm-forming ability, indicating their potential as a global health concern. 

The presence of antimicrobial residues in food is a concern and leads to economic losses, MDR *E. coli* presents a challenge as it can inhabit various environments, facilitating the acquisition or transmission of antimicrobial resistance genes [2]. Addressing the use of antimicrobials and combating emerging resistance in livestock at local and national levels requires multidisciplinary collaboration across animal, human, and environmental health. This approach, also known as “One Health”, and is essential for effective mitigation strategies [43].

## 5. Conclusions

The widespread presence of resistance to antibiotics that are critically important for human and veterinary use is an urgent concern, with several studies consistently reporting high levels of resistance towards major antibiotics. The observed multidrug resistance, especially in major *E. coli* clones, highlights the global challenge of antibiotic resistance on swine farms. Furthermore, biofilm formation, an important survival strategy of *E. coli*, exhibits significant heterogeneity among isolates from different swine farms, adding further complexity to the efforts to control these bacteria. These findings reinforce the importance of rigorous antibiotic stewardship and surveillance in swine production, as well as the need for global strategies to address the complex interactions between antibiotic resistance and biofilm formation in swine-associated *E. coli*.

## Figures and Tables

**Table 1:** Characterization of the *E. coli* isolates (n = 39) from different swine farm compartments.

*E. coli* Isolate	Farm Compartment	Antimicrobial Resistance		Phylogroup	Integrons	Virulence Factors
		Phenotype	Genotype			
AS1	Rearing	IMI-AMP-CN-S-TOB-SXT-C-TE	*sul3-cmlA-aadA1-tetA-blaTEM-ampC-ant (2)-aac(6)-Ib-sul2-blaIMP*	A	*int1-Rvint1*	*fimA-bfp*
AS2	Gestation cells	AMP-TOB-CIP-SXT-C-TE	*parC*	A	*int1-int2*	*fimA*
AS4	Maternities	IMI-AMP-AK-CN-S-TOB-CAZ-SXT-C-TE	*ant(2)-sul2*	A	*int1-int2-Rvint1*	*fimA-aer*
AS5	Gestation parks	IMI-ATM-CN-S-TOB-SXT-TE	*sul3-aadA1-tetA-blaTEM-tetM-ampC-* *ant(2)-aac(6)-Ib-sul2-blaIMP-strB*	B1	*int1-int2-Rvint1*	*fimA cnf1-aer*
AS6	Rearing	AMP-TOB-CIP-SXT-TE	*sul3-parC-qnrS-ampC-aac(6)-Ib*	D	*int1-int2-Rvint1*	*fimA-bfp-cnf1*
AS7	Gestation parks	IMI-S-TOB-SXT-TE	*aadA1-ampC-sul2-strB*	B1	*int1-Rvint1*	*fimA-bfp*
AS8	Maternities	AMP-CN-S-TOB-SXT-TE	*sul3-ampC*	A	*int1-Rvint1*	*cnf1*
AS9	Gestation cells	AMP-S-TOB-SXT-C-TE	*cmlA-aadA1-tetM-ampC-tetB-int2*	A	*int1-int2-Rvint1*	*fimA*
AS10	Gestation cells	AMP-TOB-SXT-TE	*sul3-ampC-tetB-aac(3)-IV*	A	*int1-Rvint1*	*fimA-papC-bfp-cnf1*
AS11	Maternities	IMI-ATM-AMP-S-TOB-CIP-SXT-C-TE	*sul3-cmlA-parC-blaTEM-tetB*	D	*int1-int2-Rvint1*	*fimA-cnf1*
AS12	Gestation parks	IMI-AMP-CN-S-TOB-SXT-TE	*sul3-ampC-tetB-blaIMP*	A	*int1-int2-Rvint1*	*fimA-bfp-cnf1*
AS13	Gestation parks	AMP-TOB-CIP-SXT-TE	*sul3-parC-aadA1-ant(2)-ampC*	A	*int1-Rvint1*	*fimA-bfp*
AS14	Gestation parks	AMP-CN-S-TOB-SXT-C-TE	*cmlA-blaTEM-ant(2)-*	A	*int2-Rvint1*	*-*
AS15	Gestation cells	IMI-AMP-AK-S-TOB-CIP-SXT-TE	*aac(6)-Ib-parC-aadA1-tetA-blaTEM-ampC-sul2-blaIMP*	A	*int1-int2-Rvint1*	*fimA-bfp*
AS16	Gestation cells	AMP-TOB-CIP-SXT-C-TE	*sul3-cmlA-parC-tetM-ampC*	A	*int1-Rvint1*	*fimA-bfp*
AS18	Nulliparous	IMI-AMP-S-TOB-TE	*aadA1-blaTEM-tetB-ampC-aac(6)-Ib-blaIMP-strB*	A	*int2*	*bfp*
AS19	Rearing	IMI-AMP-S-TOB-SXT-C-TE	*sul3-cmlA-aadA1-tetB-ampC-sul2-strB-aac(3)-II-aac(3)-IV*	B1	*int1*	*fimA-bfp*
AS20	Gestation parks	AMP-TOB-SXT-C-TE	*sul3-cmlA-ampC-sul2-aac(3)-II-aac(3)-IV*	B1	*int1*	*fimA-bfp*
AS21	Maternities	IMI-AMP-CN-TOB-CIP-SXT-C-TE	*sul3-parC-blaTEM-ampC-aac(6)-Ib-blaIMP-aac(3)-IV*	A	*int1*	*fimA*
AS22	Gestation cells	IMI-AUG-AMP-AK-S-TOB-SXT-TE	*sul3-aadA1-blaTEM-tetB-ampC-sul2-blaIMP-aac(3)-IV*	A	*int1-Rvint1*	*fimA*
AS23	Maternities	IMI-AUG-AMP-CN-TOB-SXT-TE	*sul3-tetA-blaTEM-ampC*	D	*int1-int2-Rvint1*	*fimA*
AS24	Maternities	AMP-S-TOB-SXT-TE	*sul3-tetA-blaTEM-tetB-aac(3)-IV*	A	*-*	*fimA*
AS25	Gestation parks	AMP-TOB-SXT-C-TE	*cmlA-blaTEM-ampC-tetB-aac(6)-Ib-aac(3)-IV*	A	*int1-Rvint1*	*fima-bfp*
AS26	Gestation cells	IMI-AUG-AMP-S-TOB-SXT-C-TE	*sul3-ampC*	A	*Rvint1*	*fima-bfp*
AS27	Gestation cells	IMI-AUG-AMP-S-TOB-SXT-C-TE	*sul3-ampC*	A	*Rvint1*	*fima-bfp*
AS28	Gestation parks	IMI-AMP-CN-S-TOB-NA-CIP-SXT-TE	*parC-tetA-blaTEM-tetB-ampC-sul2-blaIMP-strB*	A	*Rvint1*	*fimA*
AS29	Gestation parks	IMI-AMP-CN-S-TOB-NA-CIP-SXT-TE	*aadA1-tetA-blaTEM-tetB-ampC-aac(6)-Ib-sul2*	A	*int2*	*fimA*
AS30	Gestation cells	IMI-AMP-CN-S-TOB-SXT-TE	*sul3-cmlA-aadA1-tetB-blaTEM-ampC-aac(6)-Ib*	A	*rvint2-int2-Rvint1*	*fimA*
AS32	Gestation parks	IMI-AUG-AMP-AK-CN-S-TOB-CTX-CAZ-CIP-SXT-TE	*aac(6)-Ib-parC-aadA1-tetB-blaTEM-ampC-strB*	A	*rvint2-int2-Rvint1*	*papG-III*
AS35	Maternities	IMI-AMP-S-TOB-SXT-TE	*sul3-ampC-aac(3)-IV*	A	*-*	*fimA*
AS36	Rearing	IMI-AMP-AK-CN-TOB-SXT-C-TE	*teta-ant(2)-blaTEM-ampC-sul2 aac(3)-II-aac(3)-IV*	B1	*-*	*fimA*
AS37	Gestation cells	IMI-AMP-S-TOB-SXT-TE	*sul3-aadA1-tetB--blaTEM-ampC-blaIMP aac(3)-II-aac(3)-IV*	A	*int2*	*fimA-bfp*
AS38	Gestation cells	IMI-AMP-AK-S-TOB-SXT-TE	*sul3-aadA1-tetA-blaTEM-ampC-aac(3)-II-aac(3)-IV*	A	*int2-Rvint1*	*fimA-bfp*
AS39	Gestation parks	IMI-AMP-AK-CN-S-TOB-SXT-TE	*sul3-ant(2)-tetB-blaTEM-ampC-aac(3)-II-aac(3)-IV*	D	*int2*	*fimA*
AS40	Fattening	AMP-AK-TOB-C-TE	*-*	B1	*Rvint1*	*fimA*
AS41	Fattening	AMP-TOB-SXT-C-TE	*cmlA-tetM-blaTEM-ampC-aac(3)-II-aac(3)-IV*	B1	*Rvint1*	*fimA*
AS43	Gestation parks	AUG-AMP-AK-TOB-SXT-TE	*sul3-cmlA-blaTEM-ampC-aac(6)-Ib-sul2 aac(3)-II-aac(3)-IV*	B1	*Rvint1*	*fimA-bfp*
AS44	Maternities	AUG-AMP-AK-TOB-SXT-TE	*tetB-ampC-sul2 aac(3)-II*	B1	*rvint2*	*fimA-bfp*
AS45	Gestation cells	AMP-S-SXT-TE	*aadA1-tetA-blaTEM-ampC-aac(6)-Ib-sul2 aac(3)-II-aac(3)-IV*	B1	*rvint2-Rvint1*	*fimA-bfp*

**Table 2:** Characterization of the seven *E. coli* isolates chosen for the analysis of whole genome sequencing.

*E. coli* Isolate	Farm Compartment	Antimicrobial Resistance				Phylogroup	Integrons	Virulence Factors	MLST Type	O-Serotype	Plasmid Replicon
		Phenotype	Genotype	β-Lactamase Genes	Chromosomal Mutations						
AS3	Maternities	IMI-AMP-AK-CN-S-TOB-NA-CIP-SXT-C-TE	*aph(6)-Id*; *aph (3″)-Ib*; *sul2*; *tet(A*); *dfrA14*; *evgA*; *H-NS*; *acrB*; *Escherichia coli acrA*; *AcrE*; *TolC*; *emrB*; *emrR*; *mdtG*; *mdtH*; *msbA*; *marA*; *aadA*; *aadA2*; *dfrA12*; *sul2*; *cpxA*; *mdtA*; *mdtB*; *sul3*	*blaTEM-1A*	*ampC-promoter*	B1	*int1-int2*	*Cib*; *cnf1*; *csgA*; *cvaC*; *etsC*; *etsC*; *fimH*; *fyuA*; *gad*; *hlyA*; *hlyE*; *hlyF*; *hra*; *iroN*; *irp2*; *iss*; *iucC*; *iutA*; *IpfA*; *mchF*; *nipl*; *ompT*;*papAF1651A*; *papC*; *sitA*; *terC*; *traJ*; *traT*; *tsh*; *yehC*; *yehD*	ST5229	H10	*IncFIA*; *IncFIB*
AS17	Rearing	AMP-AK-S-TOB-CIP-SXT-TE	*aadA2*;*dfrA12*; *tet(M*); *tet(A*); *cmlA1*; *floR*; *evgA*; *H-NS*; *PmrF*; *acrB*; *Escherichia coli acrA*; *AcrE*; *marA*; *sul2*; *mdtH*; *cpxA*; *emrR*; *emrB*; *TolC*; *msbA*; *sul3*; *dfrA1*	*blaTEM-234*; *blaTEM-230*; *blaTEM-217*; *blaTEM-207*; *blaTEM-198*; *blaTEM-176*; *blaTEM-104*; *blaTEM-70*; *blaTEM-30*; *blaTEM-1B*	*-*	B1	*int1*	*csgA*; *fimH*; *gad*; *hlyE*; *IpfA*; *nIpl*; *fimH*; *gad*; *hlyE*; *IpfA*; *terC*; *yehA*; *yehB*; *yehc*; *yehD*	ST5757	H51	*InFIB*
AS31	Gestation cells	IMI-AMP-CN-S-TOB-SXT-C-TE	*aadA1*;*tet(A*); *evgA*; *emrK*; *emrY*; *QnrS1*; *dfrA12*; *aadA2*; *sul3*; *kdpE*; *H-NS*; *PmrF*; *YojI*; *Escherichia coli acrA*; *acrB*; *mdtP*; *mdtO*; *mdtN*; *eptA*; *msbA*; *acrD*; *emrR*; *emrA*; *emrB*; *bacA*; *TolC*; *cpxA*; *mdtH*; *mdtH*; *AcrE*; *AcrF*; *marA*; *mdtE*; *gadX*	*blaTEM-1A*; *blaLAP-*	*gyrA*; *gyrB*; *parC*; *parE*; *pmrA*;*pmrB*; *folP*; *23S*	A	*-*	*cea*; *csgA*; *fimH*; *gad*; *hlyE*; *IpfA*; *nIpl*; *terC*; *traJ*; *traT*; *yehA*; *yehB*; *yehC*; *yehD*	ST10	H43	*IncFII*; *lncY*
AS33	Maternities	IMI-AMP-AK-CN-S-TOB-CIP-SXT-TE	*aadA1*; *aph(6)-Id*; *aph (3″)-Ib*; *sul2*; *sul3*; *tet(A*); *dfrA1*; *PmrF*; *H-NS*; *evgA*; *TolC*; *AcrE*; *msbA*; *emrB*; *acrB*; *Escherichia coli acrA*; *marA*; *mdtG*; *cpxA*; *dfrA12*; *aadA2*; *aadA*; *sul3*; *QnrS1*	*blaTEM-1B*	*gyrA*; *gyrB*; *parC*; *parE*; *pmrA*;*pmrB*; *folP*; *23S*; *16rrsB*; *16S-rrsC*; *16S-rrsH*; *гpoB*	A	*int2*	*Cib*; *csgA*; *fimH*; *gad*; *hlyE*; *iss*; *IpfA*; *nIpl*; *ompT*; *terC*; *yehA*; *yehC*; *yehD*	ST1147	O162/H7	*ColpEC648*; *IncFIB*; *IncFIC(FIl*); *Incl1-lAlpha*)
AS34	Gestation cells	IMI-AMP-AK-S-TOB-NA-CIP-SXT-C-TE	*aadA2*; *aadA1*; *cmIA1*; *qnrB19*; *sul3*; *tet(A*); *dfrA12*; *H-NS*; *acrB*; *Escherichia coli acrA*; *emrR*; *emrB*; *mdtG*; *mdtH*; *evgA*; *bac*; *cpxA*; *mdtA*; *YojI*; *mdtN*; *gadX*; *mdtF*; *mdtE*; *marA*; *TolC*; *eptA*; *SAT-2*	*blaTEM-1B*	*gyrA*; *gyrB*; *parC*; *parE*; *pmrA*;*pmrB*; *folP*; *23S*; *16S-rrsB*; *16S-rrsC*; *16S-rrsH*; *гpoB*; *ampC-promoter*	A	*-*	*astA*; *csgA*; *fimH*; *gad*; *hlyE*; *nIpl*; *terC*; *traJ*; *traT*; *yehA*; *yehB*; *yehC*; *yehD*	ST48	O101/H9	*IncFIA(HI1*); *IncFIB(K*); *IncFIl*; *IncX1*
AS42	Gestation parks	AUG-AMP-AK-TOB-SXT-TE	*aadA2*; *aph(6)-Id*; *aph(3″)-Ib*; *qnrS1*; *sul3*; *tet(A*); *dfrA12*; *H-NS*; *evgA*; *mdtE*; *msbA*; *AcrE*; *emrB*; *emrR*; *acrB*; *Escherichia coli acrA*; *mdtG*; *mdtH*; *marA*; *cpxA*; *TolC*; *sul2*	*blaTEM-1B*; *blaLAP-2*	*gyrA*; *gyrB*; *parC*; *parE*; *pmrB*; *folP*; *23S*; *ampC*; *16S-rrsB*; *16S-rrsC*; *16S-rrsH*; *гpoB*; *ampC-promoter*	B1	*Rvint1*	*astA*; *csgA*; *fimH*; *fyuA*; *hlyE*; *irp2*; *kpsE*;*kpsMIll_K98*; *nIpl*; *terC*; *traJ*; *traT*; *tsh*; *yehA*; *yehB*; *yehC*;*yehD*	ST101	O13/O129/H11	*Col (MG828*); *IncFIB(K*); *IncFIB(pLF82*); *IncFIl*; *IncX1*; *p0111*
AS46	Gestation cells	AMP-AK-S-TOB-SXT-C-TE	*aadA1*; *aph(3’)-la*; *CmIA1*; *flor*; *tet(M*); *tet(A*); *mdtN*; *YojI*; *PmrF*; *acrB*; *Escherichia coli acrA*; *cpxA*; *acrD*; *emrA*; *emrB*; *mdtH*; *mdtG*; *bacA*; *TolC*; *H-NS*; *marA*; *msbA*; *dfrA12*; *aadA2*; *evgS*; *evgA*; *gadX*; *mdtF*; *mdtE*; *AcrE*; *AcrF*; *emrY*; *sul3*	*blaTEM-1B*	*gyrA*; *parC*; *parE*; *pmrA*; *pmrB*; *folP*; *23S*; *16S-rrsB*; *16S-rrsC*; *16S-rrsH*; *гpoB*; *ampC-promoter*	B1	*Rvint1*	*anr*; *csgA*; *fdeC*; *fimH*; *gad*; *hlyE*; *iss*; *IpfA*; *nIpl*; *terC*; *traJ*; *traT*; *yehA*; *yehB*; *yehC*; *yehD*	ST10	H2/H35/O128	*InFIB*

## Data Availability

No new data were created or analyzed in this study. Data are contained within the article.
